# A reactive oxygen species scoring system predicts cisplatin sensitivity and prognosis in ovarian cancer patients

**DOI:** 10.1186/s12885-019-6288-7

**Published:** 2019-11-08

**Authors:** Chaoyang Sun, Ensong Guo, Bo Zhou, Wanying Shan, Jia Huang, Danhui Weng, Peng Wu, Changyu Wang, Shixuan Wang, Wei Zhang, Qinglei Gao, Xiaoyan Xu, Beibei Wang, Junbo Hu, Ding Ma, Gang Chen

**Affiliations:** 1 Cancer Biology Research Center (Key laboratory of Chinese Ministry of Education), Tongji Hospital, Tongji Medical College, Huazhong University of Science and Technology, Wuhan, People’s Republic of China; 2 Department of Gynecology and Obstetrics, Tongji Hospital, Tongji Medical College, Huazhong University of Science and Technology, Wuhan, People’s Republic of China; 3 Department of Surgery, Tongji Hospital, Tongji Medical College, Huazhong University of Science and Technology, Wuhan, People’s Republic of China

**Keywords:** Serous ovarian Cancer, ROS, Scoring system, Prognosis

## Abstract

**Background:**

To reveal roles of reactive oxygen species (ROS) status in chemotherapy resistance and to develop a ROS scoring system for prognosis prediction in ovarian cancer.

**Methods:**

We tested the sensitizing effects of ROS elevating drugs to cisplatin (cDDP) in ovarian cancer both in vitro and in vivo. A ROS scoring system was developed using The Cancer Genome Atlas (TCGA) database of ovarian cancer. The associations between ROS scores and overall survival (OS) were analyzed in TCGA, Tothill dataset, and our in-house dataset (TJ dataset).

**Results:**

ROS-inducing drugs increased cisplatin-induced ovarian cancer cell injury in vitro and in vivo. ROS scoring system was established using 25 ROS-related genes. Patients were divided into low (scores 0–12) and high (scores 13–25) score groups. Improved patient survival was associated with higher scores (TCGA dataset hazard ratio (HR) = 0.43, *P* < 0.001; Tothill dataset HR = 0.65, *P* = 0.022; TJ dataset HR = 0.40, *P* = 0.003). The score was also significantly associated with OS in multiple datasets (TCGA dataset r^2^ = 0.574, *P* = 0.032; Thothill dataset r^2^ = 0.266, *P* = 0.049; TJ dataset r^2^ = 0.632, *P* = 0.001) and with cisplatin sensitivity in ovarian cancer cell lines (r^2^ = 0.799, *P* = 0.016) when used as a continuous variable. The scoring system showed better prognostic performance than other clinical factors by receiver operating characteristic (ROC) curves (TCGA dataset area under the curve (AUC) = 0.71 v.s. 0.65, Tothill dataset AUC = 0.73 v.s. 0.67, TJ dataset AUC = 0.74 v.s. 0.66).

**Conclusions:**

ROS status is associated with chemotherapy resistance. ROS score system might be a prognostic biomarker in predicting the survival benefit from ovarian cancer patients.

## Background

Ovarian cancer is the second common diagnosed and the most lethal of the various gynecologic malignancies [1]. Although ovarian cancer patients are sensitive to platinum- and taxane-based chemotherapy during initial treatment, a significant proportion of patients relapse and develop platinum resistance [1, 2].

Reactive oxygen species (ROS) are oxygen-containing reactive chemical molecules generated during metabolic processes. ROS play an essential role in signal transduction pathways [3], cell cycle progression [3–5], gene transcription [3, 6], cell differentiation [7, 8], and cell death [6]. Elevated oxidative stress and delicate redox balance were detected in cancer cells due to activation of oncogene, high metabolic activity, and mitochondrial malfunction [6, 9, 10]. Deprivation of the redox balance through an increase in ROS levels or a decrease in the cellular antioxidant capacity to induce cellular ROS burst have shown therapeutic benefits in cancer cells [11–13]. Most chemotherapeutics, including platinum and taxanes, exert anti-cancer effects by inducing ROS-mediated cell damage in cancer cells [14–17]. Since new therapeutic approaches combining chemotherapeutics with ROS-elevating drugs have exhibited improvement of the cytotoxicity and reduction of the resistance [18–20]. Moreover, some studies have shown that part of patients with drug resistance attribute to the lower level of tumor cell oxidative stress and stronger antioxidant ability [21, 22].

Therefore, it is of increasing interest to develop a prognostic method to predict patients who will benefit from chemotherapy or additive ROS inducer based on the quantifiable criteria of ROS status. In this paper, we reflected that ROS is involved in the drug resistance and chemo-sensitivity in vitro and in vivo. We then established a comprehensive scoring system through analyzing the relationship between the expressions of ROS pathway genes, including genes involved in oxidative stress, oxidation reaction, antioxidant response, and prognosis of patients in TCGA database. We validated the effect of this system in Tothill database and ovarian cancer patients from our hospital. The result showed that this scoring system might be clinically applied to predict the outcome of chemotherapy in ovarian cancer patients.

## Methods

### Cell culture

SKOV3 (HTB-77), Caov3 (HTB-75), OVCAR3 (HTB-161), and OV-90 (CRL-11732) ovarian cancer cell lines were purchased from the American Type Culture Collection (ATCC, Manassas, VA, US) and cultured as recommended. Caov3 was cultured in DMEM with 10% FBS (Invitrogen) and OVCAR3 was cultured in RPMI 1640 with 10% FBS (Invitrogen) and 10μg/ml insulin (Bovine). SKOV3 was cultured in McCoy 5A with 10% FBS (Invitrogen). OV-90 was grown in a 1:1 mixture of MCDB 105 medium containing a final concentration of 1.5 g/L sodium bicarbonate and Medium 199 containing a final concentration of 2.2 g/L sodium bicarbonate with 15% FBS (Thermo Scientific). Cisplatin-sensitive ovarian cancers cell line (OV2008) and its resistant variant (C13*) were gifts from Prof. Benjamin K. Tsang in the Ottawa Health Research Institute, Ottawa, Canada [23] and cultured in RPMI 1640 medium with 10% FBS (Invitrogen). All cells were free from mycoplasma and were used between 3 and 5 passages after thawing. All cells were authenticated by china center for type culture collection (CTCC, Wuhan, China) using short tandem repeat (STR) DNA profiling. Primary cell lines were isolated from ovarian cancer tissue specimens of patients undergoing surgical resection as previously described [24] and cultured in DMEM/F12 medium (Invitrogen) with 20% FBS (Invitrogen). The passage number of primary ovarian cancer cells during the experiment was ranged from 3 to 8 generations. All cell lines were cultured in a 37 °C humidified atmosphere containing 5% CO_2_.

### Assessment of cell viability

Viability of cells were assessed by Cell Counting Kit-8 reagent (CCK8, Dojindo, Tokyo, Japan). 5 × 10^3^ cancer cells were seed in 96-well plate and treated with cDDP at different concentrations for 48 h with or without ROS-elevating (PLX4032 (1 μM), Piperlongumine (PIPER, 10 μM) and β-phenylethyl isothiocyanate (PEITC, 10 μM)) or ROS-scavenging drugs (glutathione (GSH, 2 mM), N-acetyl cysteine (NAC, 1 mM) and Vitamin C (VitC, 1 mM)). Supernatants were removed and 100 μl of CCK8 solution (1:10 dilution) were added to the cancer cells. After 2 h incubation at 37 °C in dark, optical density (OD) at 450 nm was measured by a microplate reader. The IC50 value for each cell line was determined by nonlinear regression analysis using GraphPad Prism (GraphPad Software Inc., San Diego, CA). The results were tested by three independent experiments.

### ROS measurements

ROS measurement was assayed using dichloro-dihydro-fluorescein diacetate (DCFDA; Beyotime, Shanghai, China), according to the manufacturers’ instructions. Briefly, cells were loaded with DCFH-DA, washed with ice-cold HBSS. Then, the fluorescence intensity of the cells was measured at 488 nm by flow cytometry.

### Tumor Xenograft studies

The study was approved by the Ethical Committee of the Medical Faculty of Tongji Medical College (Wuhan, China), and performed in accordance with the relevant guidelines and regulations. Six-week-old athymic female homozygous BALB/c nude mice (SPF) were bought from Beijing Hua Fukang biological Polytron Technologies Inc., and reared in accordance with the relevant guidelines and regulations. C13* cells (10^7^ cells/0.1 ml PBS/mice) intraperitoneally injected into the right flank of BALB/C nude mice, under isoflurane-induced anesthesia. Treatment began when the tumor volume reached between 70 and 100mm^3^. The mice were randomly divided into 4 groups (*n* = 6) including PBS group, cDDP treatment group (cDDP 2.5 mg/kg, i.p., every 4 days for 28 days), PIPER treatment group (PIPER 2 mg/kg, i.p., daily for 28 consecutive days), and cDDP combination with PIPER treatment group (same dose as used in the single-agent groups). Following the initial treatment, the tumor sizes were measured every 2 days. Tumor volumes (V) were calculated by the following formula: V (mm^3^) = length × (square of width)/2. The mice were euthanized by cervical dislocation. Tumors were excised, weighed, paraformaldehyde-fixed paraffin embedding and used for ex vivo immunohistochemical staining.

### Immunohistochemistry (IHC) and scoring

IHC staining was performed as described previously [25]. Briefly, tissue sections were incubated with antibody γ-H2AX (Abcam, Cat: ab2893, dilution 1:200), Ki67 (Abcam, Cat: ab15580, dilution 1:200), CD34 Mouse monoclonal (Abcam, Cat: ab198395, 1:1000), and cleaved Caspase-3 (Cell signaling Technology, Cat: 9661,1:200) overnight at 4 °C and stained by 3,3′-diaminobenzidine (DAB). Tumor-cell staining was assigned a score as described previously [25]. All specimens were evaluated by two independent experts simultaneously.

### Study design, patients, and sample processing

This study was designed using a discovery stage and validation phase. In the discovery stage, 511 SOC patients with level 3 mRNA data were obtained from the TCGA database [26] to establish a scoring system. In the validation phase, the scores were validated using the largest outside independent dataset-Tothill dataset (GSE9899, *n* = 285). Patients lacking the serous pathologic type (*n* = 45) were excluded from the Tothill dataset. To further validate the scoring system, 105 blocks of formalin-fixed, paraffin-embedded (FFPE) tissues from primary epithelial ovarian cancer were obtained. The study was approved by the Ethical Committee of the Medical Faculty of Tongji Medical College. All patients written informed consents. The surgical staging was assessed in accordance with the International Federation of Gynecology and Obstetrics (FIGO) classification. Optimal debulking was defined as ≤1 cm residual disease. All clinicopathological characteristics are reported in Additional file 1: Table S1.

### Quantitative real-time PCR (qRT-PCR)

RNAs from 105 FFPE cases were extracted from four 10-μm-thick FFPE sections using the miRNeasy FFPE kit (Qiagen, Valencia, CA, USA). The cDNA was synthesized by the SuperScript® IV First-Strand Synthesis System (Thermo Fisher Scientific, China). Real-time PCR amplification was performed on an CFX Connect™ Real-Time PCR Detection System with SYBR reagent (Bio-Rad, China). GAPDH was used as an internal control.

### Statistical analysis

Student’s t-test was performed to compare the statistical difference between two groups. Multiple comparisons were accessed using the one-way analysis of variance (ANOVA). Survival was analyzed by the Kaplan–Meier method with the log-rank test. Univariate and multivariable Cox regression analyses were used to test for statistical independence between the score, pathological, and clinical variables. The relationship between the score and median OS was measured by pearson correlation coefficient. Area under the curve (AUC) values were calculated from the ROC curves. All tests were two-sided, and *P*-values < 0.05 were considered to indicate a statistically significant difference. All calculations were performed with SPSS (Version 25.0).

## Results

### ROS levels are associated with cDDP sensitivity of ovarian cancer both in vitro and in vivo

**Fig. 1:**
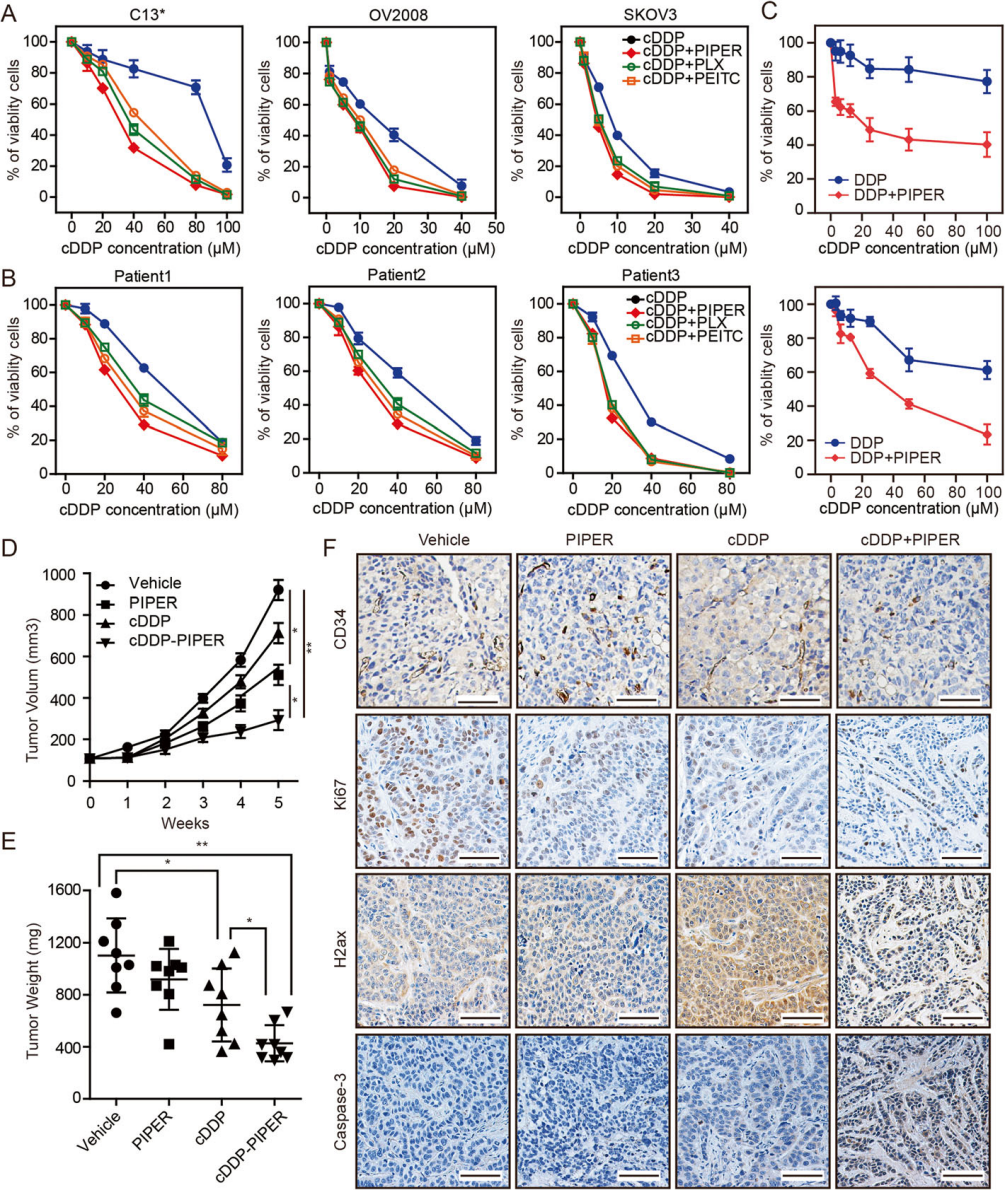
ROS levels are associated with cDDP sensitivity of ovarian cancer. **a** cDDP IC50 curves for ovarian cancer cell lines C13*, OV2008 and SKOV3 with or without ROS-elevating drugs (PLX4032, 1 μM, Piperlongumine (PIPER, 10 μM) and β-phenylethyl isothiocyanate (PEITC, 10 μM)). **b** Cell viability of 3 strains of primary cancer cells was assayed after treatment with increasing concentrations of cDDP with or without ROS-elevating drugs for 48 h by CCK-8. **c** Cell viability of primary cancer cells derived from patients with recurrent ovarian cancer or primary ovarian cancer was assayed after treatment with increasing concentrations of cDDP with or without PIPER for 48 h by CCK-8. **a**-**c** The two-tailed *P*-values < 0.05 were considered to indicate statistically significant differences. The results were tested by three independent experiments. **d** Growth curves of C13* subcutaneous xenograft tumors treated with vehicle, cDDP (2 mg/kg, intraperitoneally every 4 days), PIPER (2 mg/kg, intraperitoneally daily for 28 consecutive days), and cDDP plus PIPER (same dose as used in the single-agent groups) are shown. Tumor volumes were calculated as length × (square of width)/2. *n* = 8 per group. (*P < .05, **P < .001, two-sided Student t-test). **e** Tumor weights in nude mice were measured on day 35 after tumor cell injection. n = 8 per group. (*P < .05, **P < .001, two-sided Student t-test). (F) The immunohistochemistry analyses for caspase 3, Ki67, γ-H2AXand CD34 staining were carried out on C13* xenograft tumor sections collected from mice treated with the indicated treatments. Representative staining is shown. Scale bars = 50 μm. Data in (**a**–**e**) are the mean values ±95% confidence intervals.

To determine whether the ROS levels of ovarian cancer cells play a role in cisplatin (cDDP) resistance, 6 cell lines, including SKOV3, Caov3, OVCAR3, OV-90, OV2008, and C13* were exposed to different concentrations of cDDP (Fig. 1a and Additional file 2: Figure S1A). The IC_50_ of CDDP-treated cell lines are displayed in Table S2. We also measured the intrinsic ROS levels in each of the cell lines using flow cytometry (Additional file 2: Figure S1B). However, intrinsic ROS levels had no statistically significant impact on cDDP sensitivity. Then, the cancer cells were treated with different cDDP concentrations in addition to fixed concentrations of ROS-elevating (PLX4032 (1 μM), Piperlongumine (PIPER, 10 μM) and β-phenylethyl isothiocyanate (PEITC, 10 μM)) or ROS-scavenging drugs (glutathione (GSH, 2 mM), N-acetyl cysteine (NAC, 1 mM) and Vitamin C (VitC, 1 mM)). The doses of ROS-elevating or ROS-scavenging were preselected while mono-therapy has no effect on cancer cell proliferation or apoptosis (Additional file 2: Figure S1C). Interestingly, most ROS-elevating drugs increased cDDP cytotoxicity in all ovarian cancer cell lines, especially in C13*, OV2008 and SKOV3 (Fig. 1a, Additional file 2: Figure S1A, and Additional file 3: Table S2). By contrast, the cytotoxicity of cDDP in cell lines such as OV90 and OVCAR3 was reduced when combined with ROS-scavenging drugs (Additional file 2: Figure S1A and Additional file 3: Table S2). While Caov3 exhibited a moderate response in the presence of both ROS-scavenging and ROS-elevating drugs (Additional file 2: Figure S1A and Additional file 3: Table S2). Furthermore, the sensitizing effects of ROS-elevating drugs were confirmed in 6 primary cells derived from ovarian cancer patients (Fig. 1b, Additional file 2: Figure S1D and Additional file 3: Table S2), supporting its clinical relevance. PIPER showed a better sensitizing effect of cDDP cytotoxicity than other ROS-scavenging drugs, we further verified its sensitizing effect in another 5 primary cells. Five primary cell lines were derived from 3 patients with recurrent ovarian cancer and 2 patients with primary ovarian cancer. As expected, no matter what kind of ovarian cancer patients, PIPER increased cDDP cytotoxicity (Fig. 1c and Additional file 2: Figure S1E).

On the basis of the sensitizing effects of ROS-elevating drugs on cDDP in cell lines and primary ovarian cancer cells, we explored cDDP and PIPER combinations in C13* (cDDP resistant) xenograft tumors. As expected, C13* tumors are highly resistant to cDDP mono-therapy. Combination of PIPER and cDDP markedly delay tumor growth, while PIPER mono-therapy showed a minimal effect on tumor growth (Fig. 1d and e). IHC analysis showed that combination therapy diminished blood vessels (CD34), suppressed proliferation (Ki67) and increased DNA damage (H2ax) and apoptosis (cleaved caspase-3) compared to cDDP or PIPER mono-therapy (Fig. 1f).

These results revealed that baseline ROS levels in ovarian cancer cells measured by DCFDA do not accurately predict their sensitivity to cDDP. ROS-elevating drugs increased ovarian cancer cell sensitivity to cDDP in varying degrees. So, it is necessary to build a scoring system to assess ovarian cancer patients who may benefit from the combination of cDDP and ROS-elevating drugs.

### Establishment of the ROS scoring system in ovarian cancer patients

ROS-elevating drugs sensitized ovarian cancer cells to cDDP, which to some extent depended on intrinsic ROS levels in the cells. However, there is no reliable and convenient methods to quantified ROS status in tumors. So, we developed a ROS scoring system based on expressions of ROS related genes. To identify ROS pathway genes, we selected 179 ROS related genes according to our knowledge and works of literature. Ovarian cancer patients in TCGA (*n* = 511) were divided into two groups according to the median expression values of the 179 ROS related genes individually. 25 of 179 ROS related genes were selected whose expression levels were associated (*P* < 0.15) with the OS of ovarian cancer patients in TCGA dataset (n = 511) (Table 1 and Additional file 4: Figure S2). For each patient, if high gene expression was associated with a good prognosis, all patients with a higher expression value than the median expression obtained one point, and vice versa. The scores of all candidate genes of each patient were added to obtain a total score, which was called the ROS score.

**Table 1:** ROS-related genes were used to construct the score

Gene Symbol	*P*	Survival	Name
AKT2	0	low	V-akt murine thymoma viral oncogene homolog 2
FOSB	0.005	low	FBJ murine osteosarcoma viral oncogene homolog B
CITED4	0.009	high	Cbp/p300-interacting transactivator
CYBA	0.012	high	Cytochrome b-245, alpha polypeptide
JUNB	0.013	low	Jun B proto-oncogene
CYP27B1	0.014	high	Cytochrome P450, family 27, subfamily B, polypeptide 1
FOS	0.014	low	FBJ murine osteosarcoma viral oncogene homolog
NFIX	0.027	low	Nuclear factor I/X
TXNRD1	0.041	low	Thioredoxin reductase 1
USP14	0.054	high	Ubiquitin specific peptidase 14
RIT1	0.058	low	Ras-like without CAAX 1
KEAP1	0.0581	high	Kelch-like ECH-associated protein 1
CYP3A7	0.061	high	Cytochrome P450, family 3, subfamily A, polypeptide 7
TXN	0.07	low	Thioredoxin
GCLC	0.07	high	Glutamate-cysteine ligase, catalytic subunit
AKR7A3	0.072	low	Aldo-keto reductase family 7, member A3
JUN	0.074	low	Jun proto-oncogene
CUL3	0.076	low	Cullin 3
GSTA3	0.076	high	Glutathione S-transferase alpha 3
PMF1	0.078	high	Polyamine-modulated factor 1
PPARG	0.106	low	Peroxisome proliferator-activated receptor gamma
SOD1	0.122	high	Superoxide dismutase 1, soluble
ABCC4	0.123	high	ATP-binding cassette, sub-family C, member 4
GSTM3	0.14	low	Glutathione S-transferase mu 3 (brain)
NOX4	0.142	high	NADPH oxidase 4

NOTE: “High” indicates that gene expression above the median gene expression was associated with better overall survival; “low” indicates that gene expression above the median gene expression was associated with poor overall survival

**Fig. 2:**
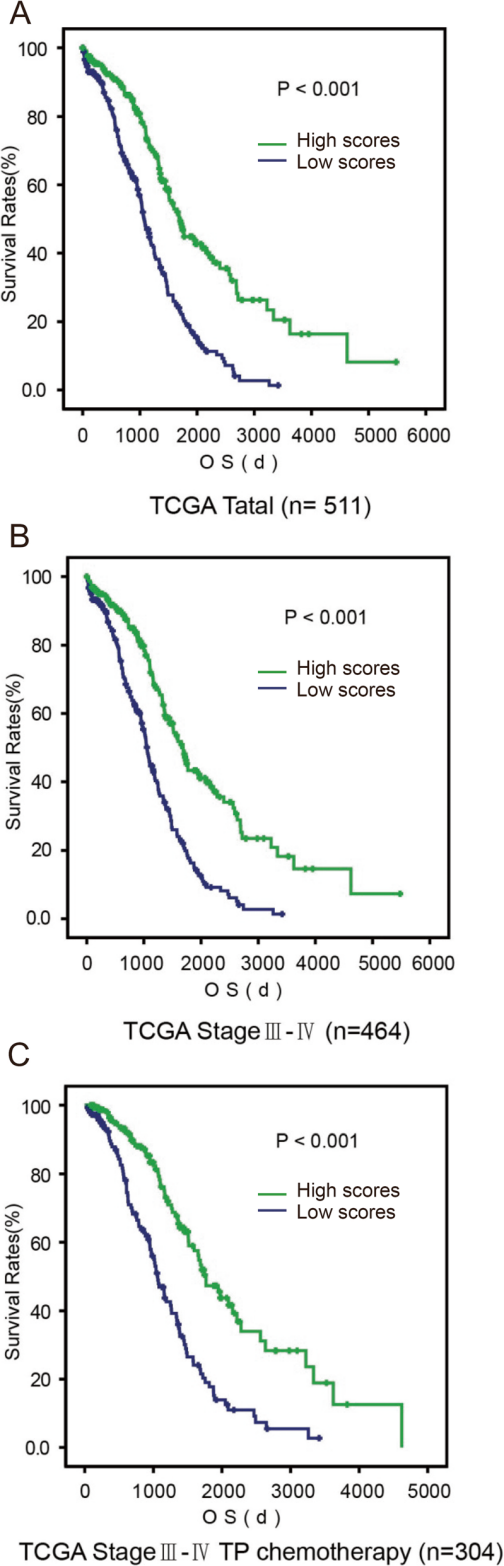
Prognostic value of the ROS scoring system in TCGA dataset. **a** A Kaplan-Meier analysis of overall survival (OS) for ovarian cancer patients in TCGA dataset with the ROS scoring system (high [scores 13–25], the green line v.s. low [scores 0–12], the blue line) is shown (*P* < 0.001, respectively, log-rank test). **b** A Kaplan-Meier analysis of overall survival (OS) for advanced stage (stage III/ IV) ovarian cancer patients in TCGA dataset with the ROS scoring system is shown (*P* < 0.001, respectively, log-rank test). **c** A Kaplan-Meier analysis of overall survival (OS) for advanced stage (stage III/ IV) ovarian cancer patients received a platinum and taxane regimen as first-line chemotherapy in TCGA dataset with the ROS scoring system is shown (*P* < 0.001, respectively, log-rank test). All statistical tests were two-sided.

The score divided patients into two categories (“low” or “high”) with equal ranges. Kaplan-Meier and univariate Cox proportional hazard regression analyses revealed patients with high scores (scores 13–25) had better OS compared to patients with low scores (scores 0–12) (high v.s. low scores, median OS = 4.66 years v.s. 2.92 years, HR = 0.43; 95% CI = 0.34 to 0.55, *P* < 0.001) (Fig. 2a). Moreover, the ROS scoring system performed well independent of tumor stages, treatments, and different molecular subtypes. Patients with high ROS scores are associated with better prognosis in stages III/IV patients (HR = 0.44; 95% CI = 0.34 to 0.56, *P* < 0.001) (Fig. 2b), in stage III/IV patients receiving first-line chemotherapy with platinum and taxane regimens (HR = 0.38; 95% CI = 0.27 to 0.53, *P* < 0.001) (Fig. 2c), or in different ovarian cancer molecular subtypes (immunoreactive, differentiated, proliferative, mesenchymal) (Additional file 5: Figure S3).

### Prognosis prediction value of the ROS scoring system in ovarian cancer patients

**Fig. 3:**
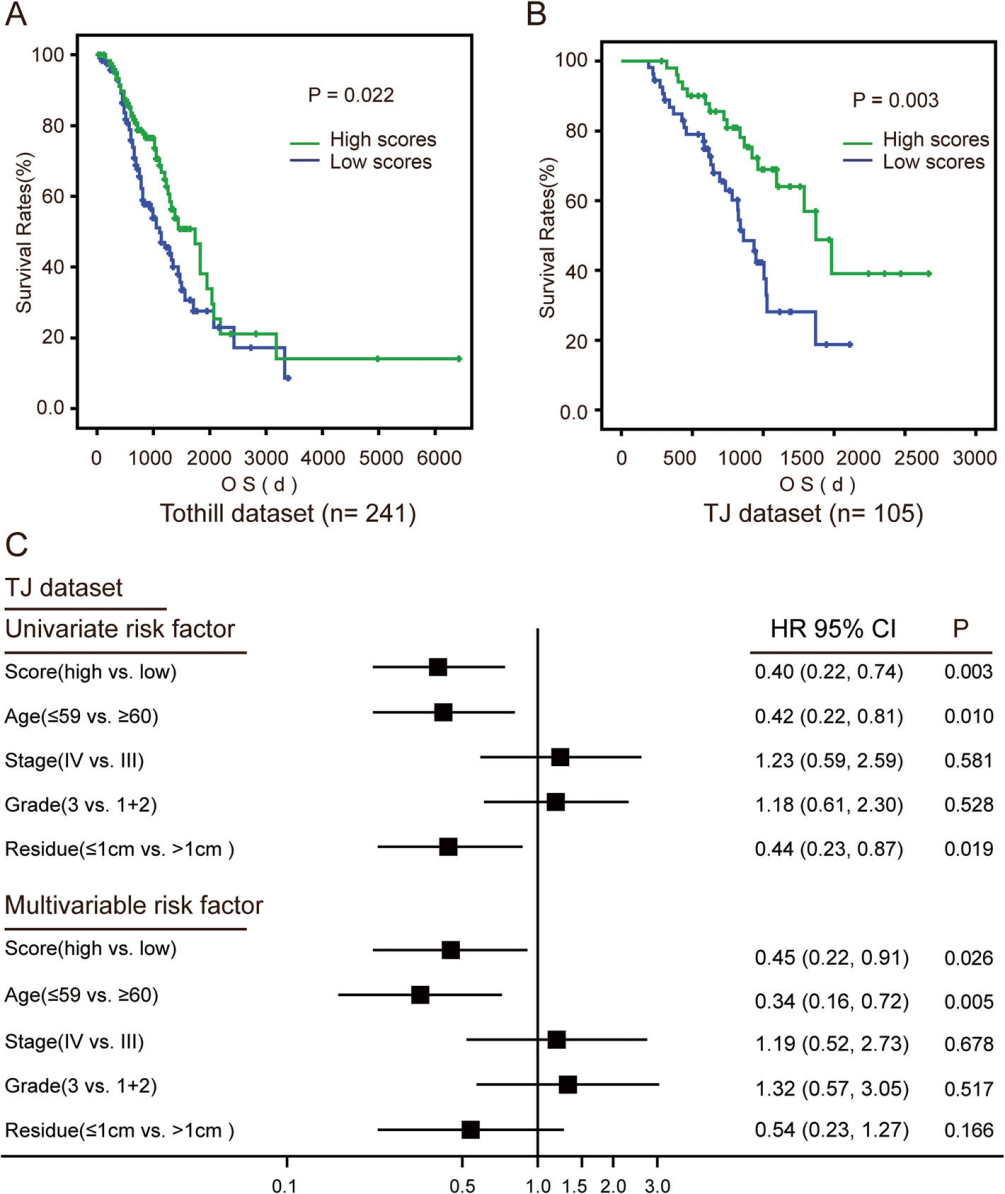
Validation of prognostic value of the ROS scoring system in Tothill and TJ datasets. **a** A Kaplan-Meier analysis of overall survival (OS) for ovarian cancer patients in Tothill (GSE9899) dataset with the ROS scoring system is shown (*P* = 0.022, respectively, log-rank test). **b** A Kaplan-Meier analysis of overall survival (OS) for ovarian cancer patients in TJ dataset with the ROS scoring system is shown (*P* = 0.003, respectively, log-rank test). **c** Univariate and multivariable Cox proportional hazards regression analyses incorporating the score and known prognostic clinical factors, including age at diagnosis (≤59 v.s. ≥60 years), stage (III v.s. IV), grade (1–2 v.s. 3), and extent of surgical debulking (0–10 v.s. ≥11 mm residual tumor); each as categorical variables. Solid squares represent the hazard ratio (HR) of death and open-ended horizontal lines represent the 95% confidence intervals (CIs). All *P* values were calculated using Cox proportional hazards analysis.

The association between ROS score and OS was confirmed in an independent outside dataset (Tothill, GSE9899), the largest ovarian cancer datasets available (high v.s. low scores, median OS = 4.77 years v.s. 3.04 years; HR = 0.65; 95% CI = 0.44 to 0.94, *P* = 0.022) (Fig. 3a). To further validate the prognosis prediction values of this ROS scoring system and broaden the scope of applications. We quantified these 25 survival-related ROS genes using a widely-applicable and convenient method, qRT-PCR analysis from FFPE tissues in 105 ovarian tumors. Consistent with afore-mentioned associations based on microarray data, patients with high ROS scores have longer survival times (high v.s. low, median OS = 3.76 years v.s. 2.36 years; HR = 0.40; 95% CI = 0.22 to 0.74, *P* = 0.002) (Fig. 3b). Univariate and multivariate Cox proportional hazard regression analyses were applied to estimate the relationship between OS and the score (high v.s. low) compared with other clinical factors, including age (≤59 v.s. ≥60 years), FIGO stage (IV v.s. III), histological grade (3 v.s. 1–2), and extent of surgical debulking (0–10 v.s. ≥11 mm residual tumor). Only the score is an independent prognostic factor in TCGA, Tothill dataset and our in house patients datasets (Fig. 3c, Additional file 5: Figure S4).

### The contribution of the ROS scoring system as a continuous variable toward prediction of OS in all datasets and cDDP sensitivity in 6 ovarian cancer cell lines

**Fig. 4:**
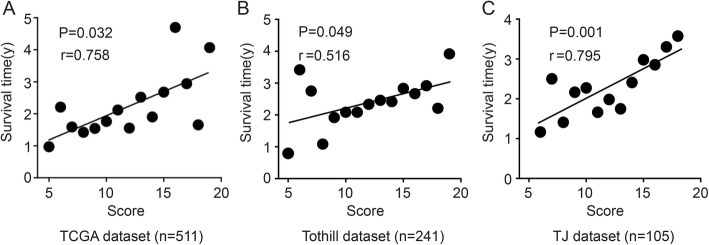
Prognostic value of the ROS scoring system in all datasets. **a**-**c** Correlation of score as a continuous variable with OS in TCGA (**a**), Tothill (**b**) and TJ (**c**) datasets. For each patient’s tumor, a point was given for each ROS related gene for which higher than median expression was associated with longer survival, and vice versa. The sum of these points constituted our score.

To further verify the associations between scores and patients’ survival, we performed correlation analysis between each score and the median survival times in patients with the same scores. Interestingly, there was a positive correlation between scores and the median survival times (r = 0.758, *P* = 0.032 in TCGA dataset; r = 0.516, *P* = 0.049 in Tothill dataset; and r = 0.795, *P* = 0.001in TJ dataset) (Fig. 4).

Univariate and multivariate analyses were repeated when the ROS score was assessed as a continuous variable. Again, the ROS score outperformed other clinical covariates as an independent prognostic factor in TCGA dataset, Tothill dataset, and in TJ dataset (Table 2).

**Table 2:** Univariate and multivariable analysis using prognostic factors in all of datasets

Cohort	Characteristics	Univariate Cox Regression			Multivariate Cox Regression		
		HR	95% CI	*P*	HR	95% CI	*P*
TCGA	Score	0.878	(0.849,0.908)	2.23*10^*−14*^	0.889	(0.857,0.922)	3.27*10^*−10*^
	Age	1.019	(1.008,1.030)	*0.001*	1.018	(1.007,1.030)	*0.002*
	Grade						
	1	1		0.173	1		0.654
	2 vs 1	1.254	(0.302,5.21)	0.756	0.814	(0.191,3.459)	0.78
	3 vs 1	1.773	(0.44,7.138)	0.421	1.319	(0.325,5.358)	0.699
	Others	2.304	(0.489,10.866)	0.291	1.256	(0.252,6.25)	0.781
	Stage						
	I-II	1		*0.002*	1		*0.047*
	III	2.374	(1.258,4.479)	*0.008*	2.226	(1.032,4.802)	*0.041*
	IV	3.219	(1.633,6.345)	*0.001*	2.805	(1.241,6.336)	*0.013*
	Surgical debulking	1.293	(0.989,1.691)	0.061	1.138	(0.861,1.504)	0.362
Tothill	Score	0.929	(0.877,0.985)	*0.013*	0.924	(0.866,0.985)	*0.015*
	Age	1.021	(1.000,1.042)	*0.047*	1.025	(1.003,1.047)	*0.027*
	Grade						
	1	1		0.467	1		0.804
	2 vs 1	1.964	(0.549,6.499)	0.269	1.269	(0.374, 4.309)	0.703
	3 vs 1	2.116	(0.660,6.791)	0.208	1.246	(0.369,4.200)	0.723
	Others	0.751	(0.077,7.287)	0.805	0.47	(0.045,4.868)	0.527
	Stage						
	I-II	1		*0.01*	1		*0.046*
	III	4.012	(1.269,12.685)	*0.018*	3.319	(1.006,10.950)	*0.049*
	IV	6.657	(1.906,23.245)	*0.003*	5.118	(1.377,19.024)	*0.015*
	Surgical debulking	0.608	(0.397,0.932)	*0.022*	0.688	(0.439,1.079)	0.104
TJ	Score	0.841	(0.746,0.949)	*0.005*	0.862	(0.750,0.990)	*0.036*
	Age	1.048	(1.011,1.087)	*0.011*	1.050	(1.013,1.089)	*0.008*
	Grade						
	1	1		0.943	1		0.693
	2 vs 1	1.146	(0.363,3.622)	0.816	1.551	(0.470,5.126)	0.471
	3 vs 1	1.274	(0.496,3.272)	0.615	1.799	(0.638,5.075)	0.267
	Others	1.538	(0.296,7.981)	0.608	2.400	(0.408,14.115)	0.333
	Stage						
	I-II	1		*0.087*	1		*0.038*
	III	3.101	(1.063,9.046)	*0.038*	3.986	(1.282,12.392)	*0.017*
	IV	3.661	(1.098,12.210)	*0.035*	4.956	(1.378,17.829)	*0.014*
	Surgical debulking	0.443	(0.225,0.874)	*0.019*	0.617	(0.298,1.276)	0.193

### Predictive accuracy of the ROS scoring system in ovarian Cancer patients

To further evaluate the contribution of the score to OS prediction, ROC curve analysis was performed using the following variables: clinical covariates (age, grade, stage, and residual tumor (AGSR)); ROS score (Score); and clinical covariates plus ROS score (AGSR + Score).

**Fig. 5:**
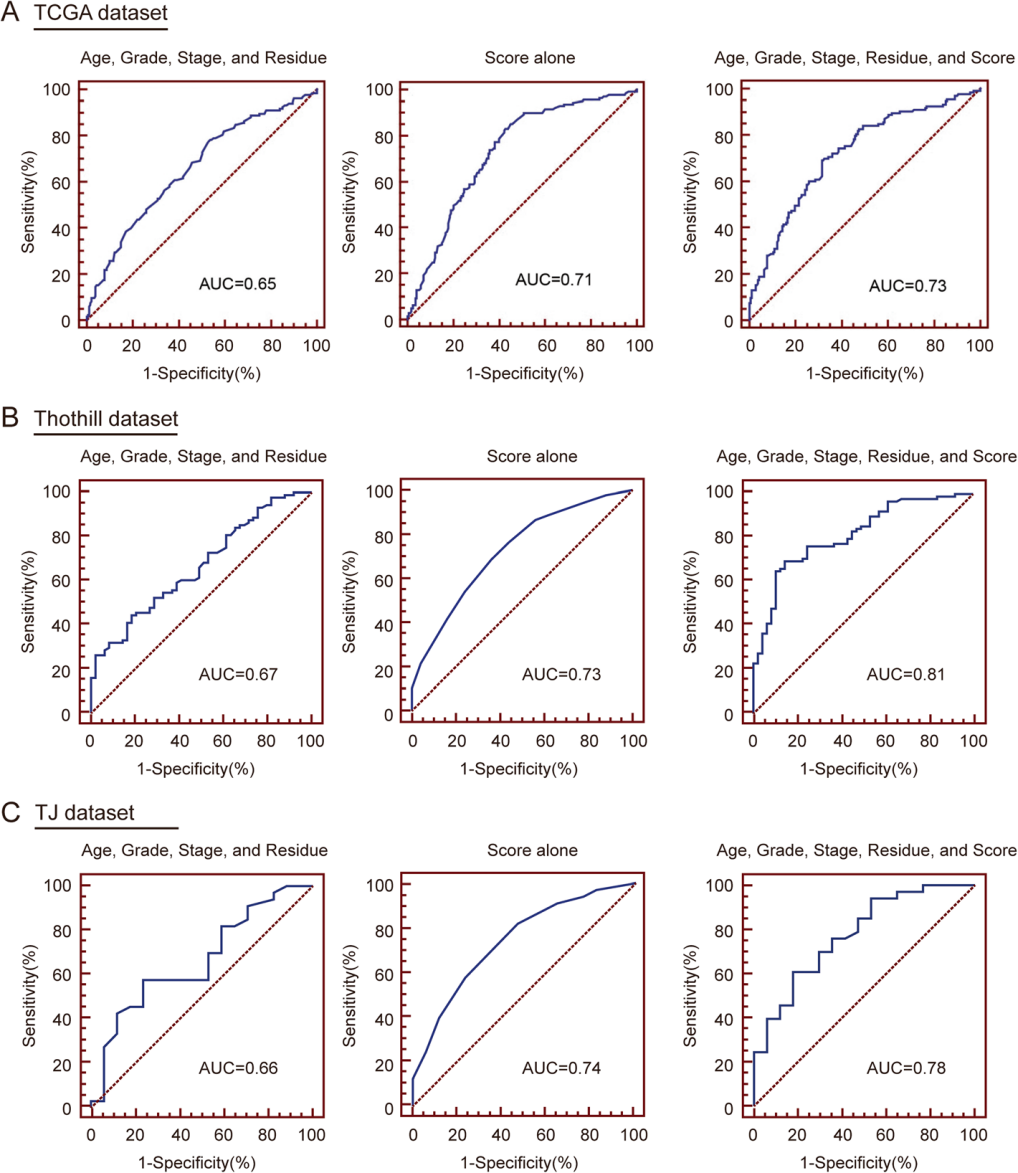
Predictive accuracy of the ROS scoring system compared with prognostic clinical factors. Receiver operating characteristic (ROC) analysis of the score and clinical covariates in predicting overall survival in TCGA (**a**), Tothill (**b**) and TJ (**c**) datasets. Using statistical models constructed based on multivariable Cox proportional hazards, ROC curves were calculated incorporating clinical variables of age, grade, and stage (left); age, grade, stage, and score (middle); and score alone (right). The area under the curve (AUC) was calculated for ROC curves, and sensitivity and specificity was calculated to assess the score performance.

For these analyses, patients were divided into two groups with a survival time higher or lower than the median OS (TCGA dataset median OS =3.79 years, Tothill dataset median OS = 3.62 years, TJ dataset median OS = 2.81 years), and those with a survival time shorter than the median OS at last follow-up were excluded. Interestedly, the score alone have higher predictive values than clinical covariates in all datasets (TCGA dataset AUC = 0.71 v.s. 0.65, Tothill dataset AUC = 0.73 v.s. 0.67, TJ dataset AUC = 0.74 v.s. 0.66) (Fig. 5a). Moreover, combined with clinical covariates and ROS score could further improve predictive performance in all dataset (TCGA dataset AUC = 0.73, Tothill dataset AUC = 0.81, TJ dataset AUC = 0.78) (Fig. 5b and c).

## Discussion

Compared with other types of cancers, one unique feature of ovarian cancer is that over 50% of ovarian cancers contain p53 mutation [27]. Specifically, p53 mutation was identified in 96% of all serous ovarian tumors [28]. Suppression of p53 led to significant decreases in the expression of SESN1, SESN2, and GPX1, suggesting that p53 is involved in cellular metabolism and antioxidant response [29–31]. So, p53 mutation could increase ROS levels and oxidative damage of DNA in ovarian cancer cells. Thus, alterations in the expression of ROS genes that affect ROS production or scavenging may be closely linked to the resistance of ovarian cancer cells to chemotherapy.

An increasing number of studies have identified relationships between ROS related genes (such as SOD, CAT, GLS2 and so on) and drug resistant [32, 33]. We found that ROS pathway function or activity plays a crucial role in chemotherapy responses in ovarian cancer cells and transplanted mouse models. Our results suggest that ROS related gene expression changes are important mechanisms by which ovarian cancer cells acquire resistance to anticancer drugs, and these changes result in different outcomes and prognoses in ovarian cancer patients.

In this study, we established a quantifiable ROS scoring system able to predict ovarian cancer patient prognosis based on the expression levels of ROS related genes in the TCGA dataset (*n* = 511). Moreover, we validated this system in another published dataset (Tothill dataset, GSE9899, *n* = 241). We further validated the scoring system in our in-house patient dataset (TJ dataset, *n* = 105). We indicated that the scoring system accurately determined the prognosis of ovarian cancer patients. The use of FFPE sections and qPCR also extended the use of the scoring system. Both datasets demonstrated that the system is prognostic for survival.

A number of gene profile-based prognosis techniques, used in combination with microarrays or PCR, have been previously developed to predict survival in patients with ovarian cancer [34–36], but the results have not been satisfactory. We demonstrated that our scoring system is superior to other known clinical factors in predicting OS, not only in the TCGA dataset but also in our dataset and another online validation set. TCGA divided ovarian cancer into four molecular subtypes (immunoreactive, differentiated, proliferative, mesenchymal) based on gene clustering, but these clusters did not associate with OS. However, statistical significance was observed when the score was applied to all subtypes except the proliferative subtype. Our score extends application of the TCGA classification model. Our system was also able to predict outcomes to first-line platinum and taxane chemotherapy in ovarian cancer. This feature has profound clinical significance because there are no other good clinical factors to predict the response to platinum-based standard chemotherapy. Most patients with advanced serous ovarian cancer will relapse after a few years even after standard therapies like thorough operation and chemotherapy are used. In addition, about 30% of patients with primary platinum resistance undergo multiple cycles of useless and potentially toxic treatment before second-line drug treatments are used. Moreover, agents that increase the ROS levels of cancer cells could be used as a standard treatment to improve chemotherapeutic responses for patients with ovarian cancers with low ROS levels.

In this study, we just demonstrate the prognostic value of the ROS scoring system, which leads to the possibility of clinical application. For individual ovarian cancer patient with poor prognosis predicted by the ROS scoring system, if possible, we can combine ROS-inducing drugs with platinum- and taxane-based chemotherapies to improve outcomes. We know there are lots of problems, such as the in vivo stability of ROS-elevating drugs, targeted property, and safety need to be resolved before ROS becoming a therapeutic target. However, further study based on patient-derived tumor xenograft (PDX) animal models for intraperitoneal administration of ROS-elevating drugs may lead to the possibility of clinical transformation.

This study has several limitations. First, although we reproduced our findings in two other datasets, this study is a retrospective analysis, and sample selection bias may exist. Of course, we hope that this score will be tested prospectively in a clinical trial, and we believe that the score is ready for such testing. Second, our gene expression profiling and analysis is only limited to ROS related pathways. However, other gene expression pathways that may be important in survival predictions were neglected. Third, this study is limited in its gene expression profiling. Other mechanisms of gene regulation, including microRNAs, DNA methylation, and CNV region changes were not considered. We are looking forward to future studies of this type and the development of more comprehensive prediction models.

## Conclusions

We established a ROS scoring system that could predict the outcomes of ovarian cancer patients. This system offers considerable improvement over existing methods for prognostic classification and has the potential to provide clinicians with useful, readily available information for personalized chemotherapy in the future.

## Supplementary information

**Additional file 1: Table S1.** Clinicopathologic characteristics of ovarian cancer patients in 3 datasets. (docx 16.8 kb) (DOCX 16 kb)

**Additional file 2: Figure S1.** ROS level is related to the survival of ovarian cancer cells treated with cDDP. (jpg 2.83mb) (A) Cell viability of ovarian cancer cell lines Caov3, OV90 and OVCAR3 was measured after treatment with gradient concentrations of cDDP with or without ROS-elevating (PLX4032, 1 μM, Piperlongumine (PIPER, 10 μM) and β-phenylethyl isothiocyanate (PEITC, 10 μM)) or scavenging drugs (glutathione (GSH, 2 mM), N-acetyl cysteine (NAC, 1 mM) and Vitamin C (VitC, 1 mM)) for 48 h by CCK-8. (B) Intracellular ROS concentrations of SKOV3, Caov3, OVCAR3, OV-90, OV2008, and C13* were measured by DCF-DA staining. (C) CCK8 detected cell viability of ovarian cancer cell lines after treatment with ROS-elevating. (D) cDDP IC_50_ curves for 3 strains of primary cancer cells with or without ROS-elevating or scavenging drugs for 48 h by CCK-8. (E) Cell viability of primary cancer cells derived from patients with recurrent or primary ovarian cancer was measured after treatment with gradient concentrations of cDDP with or without PIPER for 48 h by CCK-8. The two-tailed *P*-values < 0.05 were considered to indicate statistically significant differences. The results were tested by three independent experiments. (JPG 2907 kb)

**Additional file 3: Table S2.** The IC50 of ovarian cancer cell lines and primary cancer cells treated with different combinations of drugs. (docx 18.8 kb) (DOCX 18 kb)

**Additional file 4: Figure S2.** 84 of 179 ROS related genes with Kaplan-Meier log-rank *P* values < 0.5. (tif 328 kb) Genes with *P* < 0.15 are highlighted in red. (TIF 328 kb)

**Additional file 5: Figure S3.** Prognostic Value of the ROS Scoring System in four ovarian cancer molecular subtypes of TCGA. (tif 495 kb) A Kaplan-Meier analysis of overall survival (OS) were performed in the differentiated, immunoreactive, mesenchymal, and proliferative for ovarian cancer patients in TCGA dataset with the ROS scoring system (high [scores 13–25], the green line v.s. low [scores 0–12], the blue line) is shown (*P* < 0.001, log-rank test). All statistical tests were two-sided. (TIF 495 kb)

**Additional file 6: Figure S4.** Univariate analyses were performed in TCGA and Tothill dataset. (tif 1.21mb) Univariate analyses incorporating the score and known prognostic clinical factors, including age at diagnosis (≤59 v.s. ≥60 years), stage (III v.s. IV), grade (1–2 v.s. 3), and surgical debulking (0–10 v.s. ≥11 mm residual tumor). Solid squares represent the hazard ratio (HR) of death and open-ended horizontal lines represent the 95% confidence intervals (CIs). All *P* values were calculated using Cox proportional hazards analysis. (TIF 1241 kb)

**Additional file 7.** ARRIVE checklist. (PDF 397 kb)

## Data Availability

The datasets used and/or analyzed during the current study are available from the corresponding author on reasonable request.

## Abbreviations

cDDP: Cisplatin
FFPE: Formalin-fixed, paraffin-embedded
GSH: Glutathione
NAC: N-acetyl cysteine
OS: Overall survival
PEITC: β-phenylethyl isothiocyanate
PIPER: Piperlongumine
ROS: Reactive oxygen species
TCGA: The Cancer Genome Atlas
VitC: Vitamin C
FIGO: International Federation of Gynecology and Obstetrics
AUC: Area under the curve.
