# Correction to: A reactive oxygen species scoring system predicts cisplatin sensitivity and prognosis in ovarian cancer patients

**DOI:** 10.1186/s12885-020-6691-0

**Published:** 2020-03-12

**Authors:** Chaoyang Sun, Ensong Guo, Bo Zhou, Wanying Shan, Jia Huang, Danhui Weng, Peng Wu, Changyu Wang, Shixuan Wang, Wei Zhang, Qinglei Gao, Xiaoyan Xu, Beibei Wang, Junbo Hu, Ding Ma, Gang Chen

**Affiliations:** 1 Cancer Biology Research Center (Key laboratory of Chinese Ministry of Education), Tongji Hospital, Tongji Medical College, Huazhong University of Science and Technology, Wuhan, People’s Republic of China; 2 Department of Gynecology and Obstetrics, Tongji Hospital, Tongji Medical College, Huazhong University of Science and Technology, Wuhan, People’s Republic of China; 3 Department of Surgery, Tongji Hospital, Tongji Medical College, Huazhong University of Science and Technology, Wuhan, People’s Republic of China

**Correction to: BMC Cancer**

**https://doi.org/10.1186/s12885-019-6288-7**

Following publication of the original article [1], the authors reported an error in Fig. 1f.

The representative image of CD34 in cDDP+PIPER tumors (Fig. 1f, at the top-right corner) was inadvertently used incorrectly during the assembly and formatting of the images. The corrected Fig. 1 is presented in this correction article. The authors apologise for the error.

**Fig. 1:**
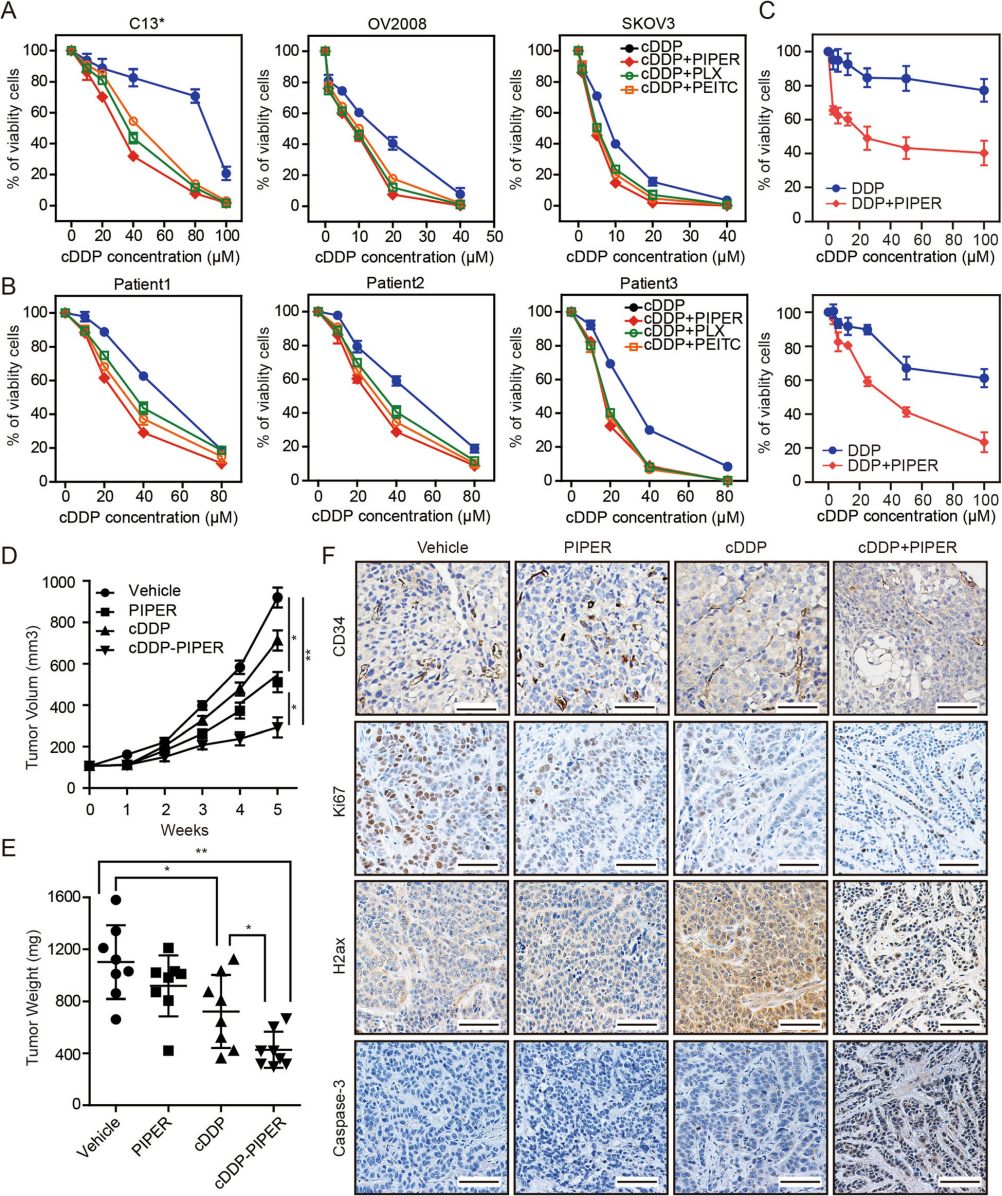
ROS levels are associated with cDDP sensitivity of ovarian cancer. **a** cDDP IC50 curves for ovarian cancer cell lines C13*, OV2008 and SKOV3 with or without ROS-elevating drugs (PLX4032, 1 μM, Piperlongumine (PIPER, 10 μM) and β-phenylethyl isothiocyanate (PEITC, 10 μM)). **b** Cell viability of 3 strains of primary cancer cells was assayed after treatment with increasing concentrations of cDDP with or without ROS-elevating drugs for 48 h by CCK-8. **c** Cell viability of primary cancer cells derived from patients with recurrent ovarian cancer or primary ovarian cancer was assayed after treatment with increasing concentrations of cDDP with or without PIPER for 48 h by CCK-8. **a**-**c** The two-tailed *P*-values < 0.05 were considered to indicate statistically significant differences. The results were tested by three independent experiments. **d** Growth curves of C13* subcutaneous xenograft tumors treated with vehicle, cDDP (2 mg/kg, intraperitoneally every 4 days), PIPER (2 mg/kg, intraperitoneally daily for 28 consecutive days), and cDDP plus PIPER (same dose as used in the single-agent groups) are shown. Tumor volumes were calculated as length × (square of width)/2. *n* = 8 per group. (*P < .05, **P < .001, two-sided Student t-test). **e** Tumor weights in nude mice were measured on day 35 after tumor cell injection. *n* = 8 per group. (*P < .05, **P < .001, two-sided Student t-test). **f** The immunohistochemistry analyses for caspase 3, Ki67, γ-H2AXand CD34 staining were carried out on C13* xenograft tumor sections collected from mice treated with the indicated treatments. Representative staining is shown. Scale bars = 50 μm. Data in (**a**–**e**) are the mean values ±95% confidence intervals
