# Supplementary material for: A reactive oxygen species scoring system predicts cisplatin sensitivity and prognosis in ovarian cancer patients
Source: BMC Cancer. 2019 Nov 8;19:1061. doi: 10.1186/s12885-019-6288-7 (PMC6839150; doi:10.1186/s12885-019-6288-7)
Supplement: Supplementary file 1 — Additional file 1: Table S1. Clinicopathologic characteristics of ovarian cancer patients in 3 datasets. (docx 16.8 kb) (DOCX 16 kb) [file 12885_2019_6288_MOESM1_ESM.docx]

Table S1

Clinicopathologic characteristics of ovarian cancer patients in 3 datasets

| **Characteristic** | **TCGA Total (n = 511)** | | **Tothill Total (n = 240)** | | **TJ Total (n = 105)** | |
| --- | --- | --- | --- | --- | --- | --- |
|  | No. of patients | Median OS (95% CI), y | No. of patients | Median OS (95% CI), y | No. of patients | Median OS (95% CI), y |
| **Age, y** |  |  |  |  |  |  |
| ≤59 | 262 | 4.1 (3.7 to 4.5) | 119 | 2.0 (2.0 to 2.6) | 82 | 2.3 (2.2 to 2.7) |
| ≥60 | 242 | 3.2 (2.8 to 3.5) | 121 | 2.7 (2.7 to 3.5) | 23 | 1.6 (1.4 to 2.1) |
| **Stage** |  |  |  |  |  |  |
| I | 15 | 6.7 (6.7- ) | 11 | 2.8 (1.9 to 3.5) | 3 | 5.1 (1.8 to 7.9) |
| II | 23 | 5.9 (3.7- ) | 10 | 3.0 (1.0 to 7.8) | 8 | 3.3 (2.8 to 4.4) |
| III | 383 | 3.7 (3.3-4.0) | 197 | 2.3 (2.4 to 2.9) | 78 | 2.0 (1.9 to 2.3) |
| IV | 81 | 2.7 (2.2- 4.2) | 21 | 2.0 (1.6 to 3.1) | 16 | 2.3 (1.6 to 2.7) |
| unkown | — | — | 1 | — | — | — |
| **Grade** |  |  |  |  |  |  |
| 1 | 5 | 5.4(4.5 to 6.2) | 7 | 4.0 (-0.1 to 10.6) | 13 | 2.3 (1.8 to 3.1) |
| 2 | 61 | 4.6(3.5 to 6.8) | 87 | 2.3 (2.1 to 2.7) | 19 | 2.2 (1.7 to 2.5) |
| 3 | 428 | 3.5(3.2 to 4.0) | 144 | 2.3 (2.4 to 3.0) | 69 | 2.2 (2.0 to 2.6) |
| unkown | — | — | 2 | — | 4 | 2.0 (1.5 to 3.0) |
| **Surgical debulking** |  |  |  |  |  |  |
| 0–10mm | 329 | 3.7(3.4 to 4.0) | 129 | 2.4 (2.4 to 3.1) | 42 | 2.4 (2.2 to 2.9) |
| ≥11mm | 128 | 3.0(2.6 to 3.5) | 65 | 2.1 (2.0 to 3.0) | 50 | 2.1 (1.9 to 2.4) |
| unkown | — | — | 46 | 2.3 (2.2 to 3.4) | 13 | 1.7 (1.2 to 2.5) |

NOTE: CI = confidence interval; OS = overall survival; — = not applicable.
