# Supplementary figures and images for: A reactive oxygen species scoring system predicts cisplatin sensitivity and prognosis in ovarian cancer patients
Source: BMC Cancer. 2019 Nov 8;19:1061. doi: 10.1186/s12885-019-6288-7 (PMC6839150; doi:10.1186/s12885-019-6288-7)

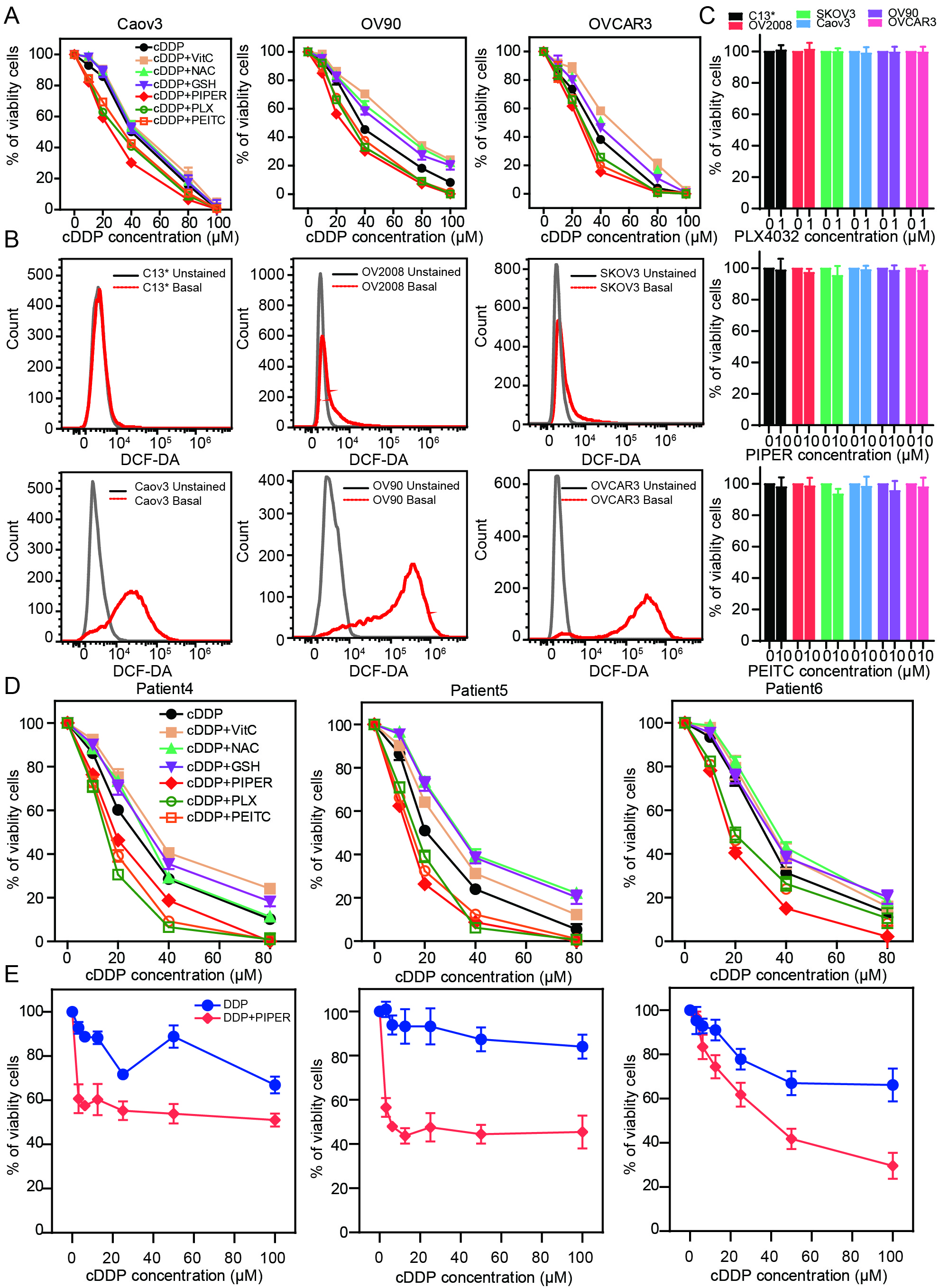

Supplement: Supplementary file 2 — Additional file 2: Figure S1. ROS level is related to the survival of ovarian cancer cells treated with cDDP. (jpg 2.83mb) (A) Cell viability of ovarian cancer cell lines Caov3, OV90 and OVCAR3 was measured after treatment with gradient concentrations of cDDP with or without ROS-elevating (PLX4032, 1 μM, Piperlongumine (PIPER, 10 μM) and β-phenylethyl isothiocyanate (PEITC, 10 μM)) or scavenging drugs (glutathione (GSH, 2 mM), N-acetyl cysteine (NAC, 1 mM) and Vitamin C (VitC, 1 mM)) for 48 h by CCK-8. (B) Intracellular ROS concentrations of SKOV3, Caov3, OVCAR3, OV-90, OV2008, and C13* were measured by DCF-DA staining. (C) CCK8 detected cell viability of ovarian cancer cell lines after treatment with ROS-elevating. (D) cDDP IC50 curves for 3 strains of primary cancer cells with or without ROS-elevating or scavenging drugs for 48 h by CCK-8. (E) Cell viability of primary cancer cells derived from patients with recurrent or primary ovarian cancer was measured after treatment with gradient concentrations of cDDP with or without PIPER for 48 h by CCK-8. The two-tailed P-values < 0.05 were considered to indicate statistically significant differences. The results were tested by three independent experiments. (JPG 2907 kb) [file 12885_2019_6288_MOESM2_ESM.jpg]

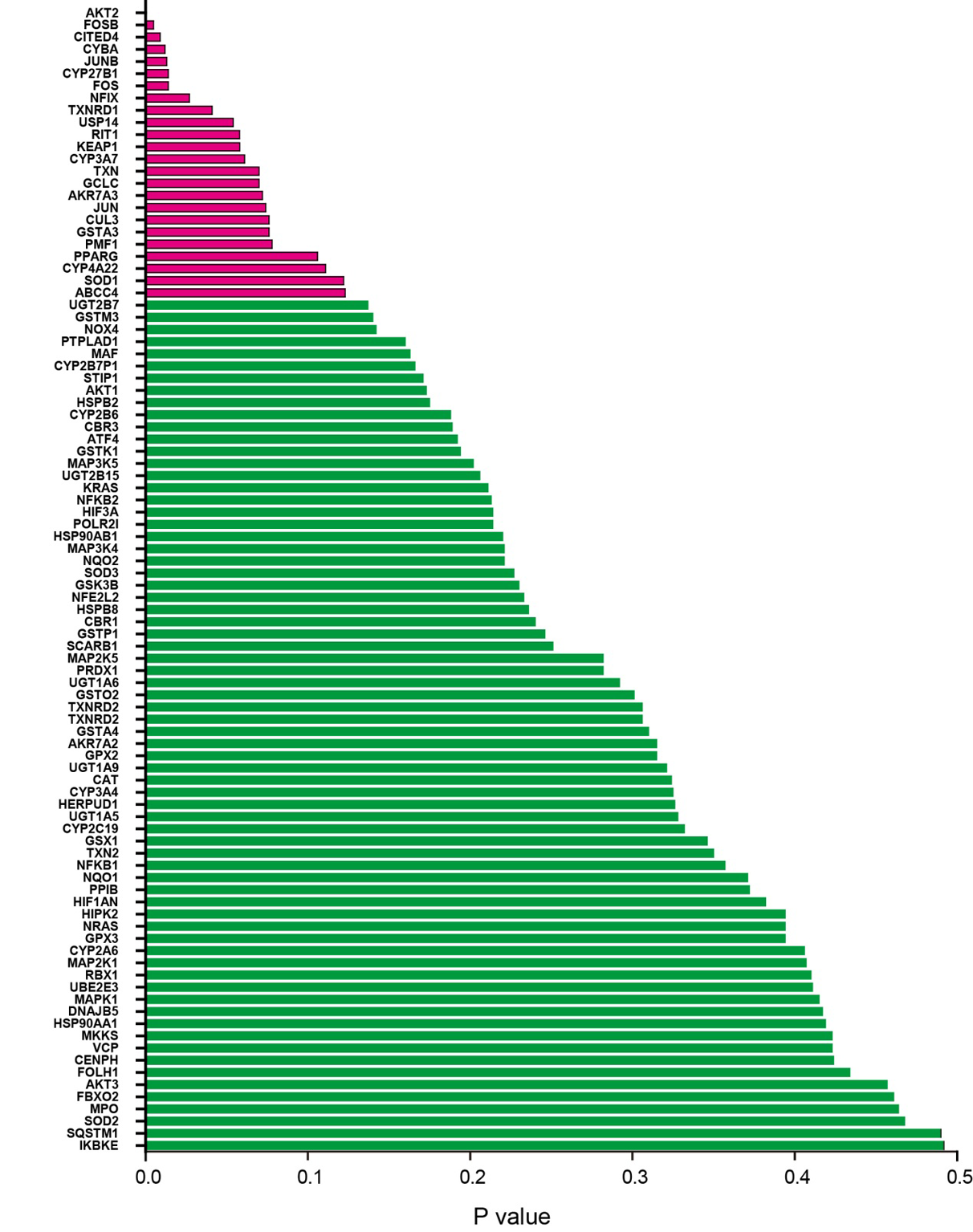

Supplement: Supplementary file 4 — Additional file 4: Figure S2. 84 of 179 ROS related genes with Kaplan-Meier log-rank P values < 0.5. (tif 328 kb) Genes with P < 0.15 are highlighted in red. (TIF 328 kb) [file 12885_2019_6288_MOESM4_ESM.tif]

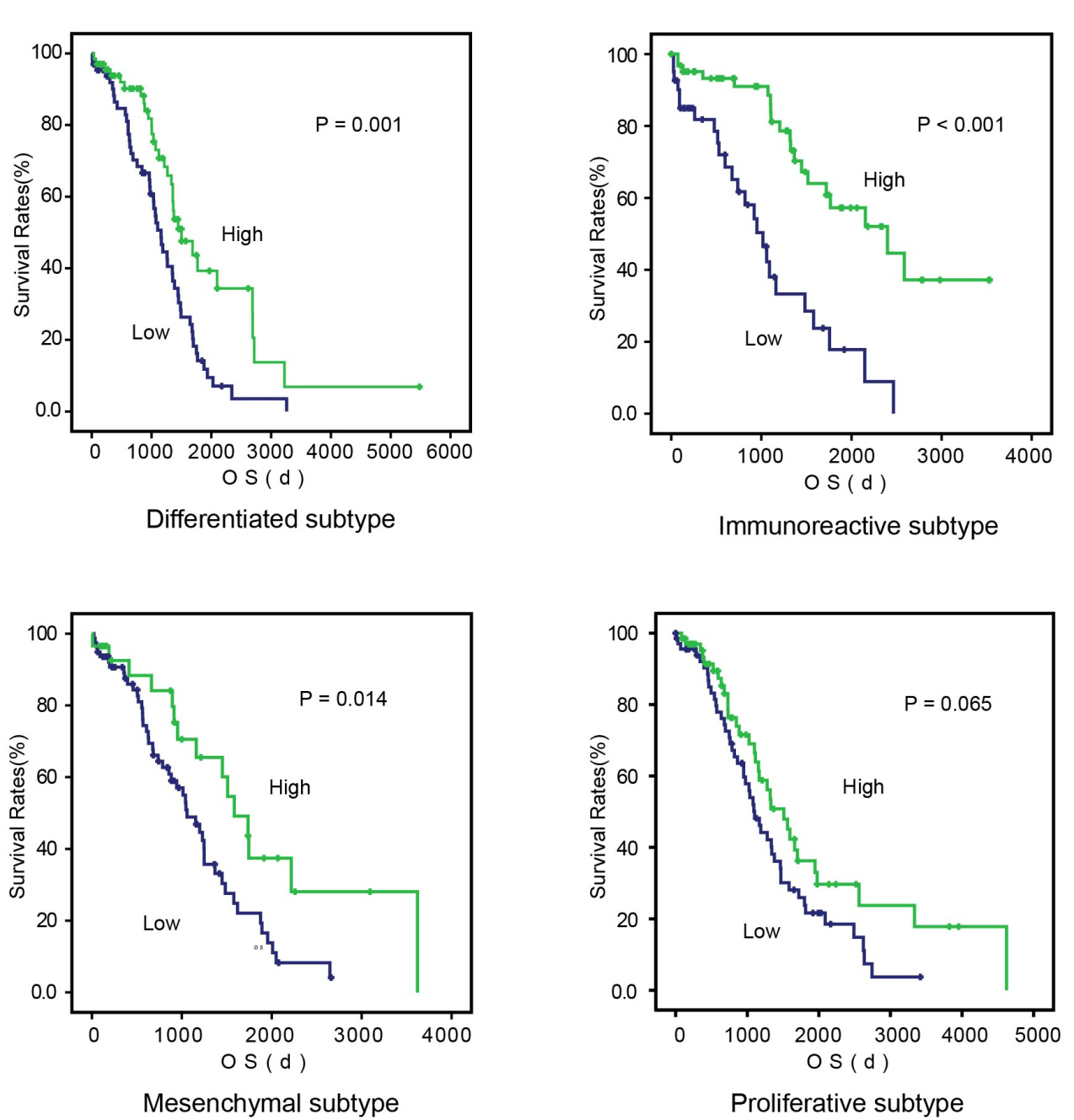

Supplement: Supplementary file 5 — Additional file 5: Figure S3. Prognostic Value of the ROS Scoring System in four ovarian cancer molecular subtypes of TCGA. (tif 495 kb) A Kaplan-Meier analysis of overall survival (OS) were performed in the differentiated, immunoreactive, mesenchymal, and proliferative for ovarian cancer patients in TCGA dataset with the ROS scoring system (high [scores 13–25], the green line v.s. low [scores 0–12], the blue line) is shown (P < 0.001, log-rank test). All statistical tests were two-sided. (TIF 495 kb) [file 12885_2019_6288_MOESM5_ESM.tif]

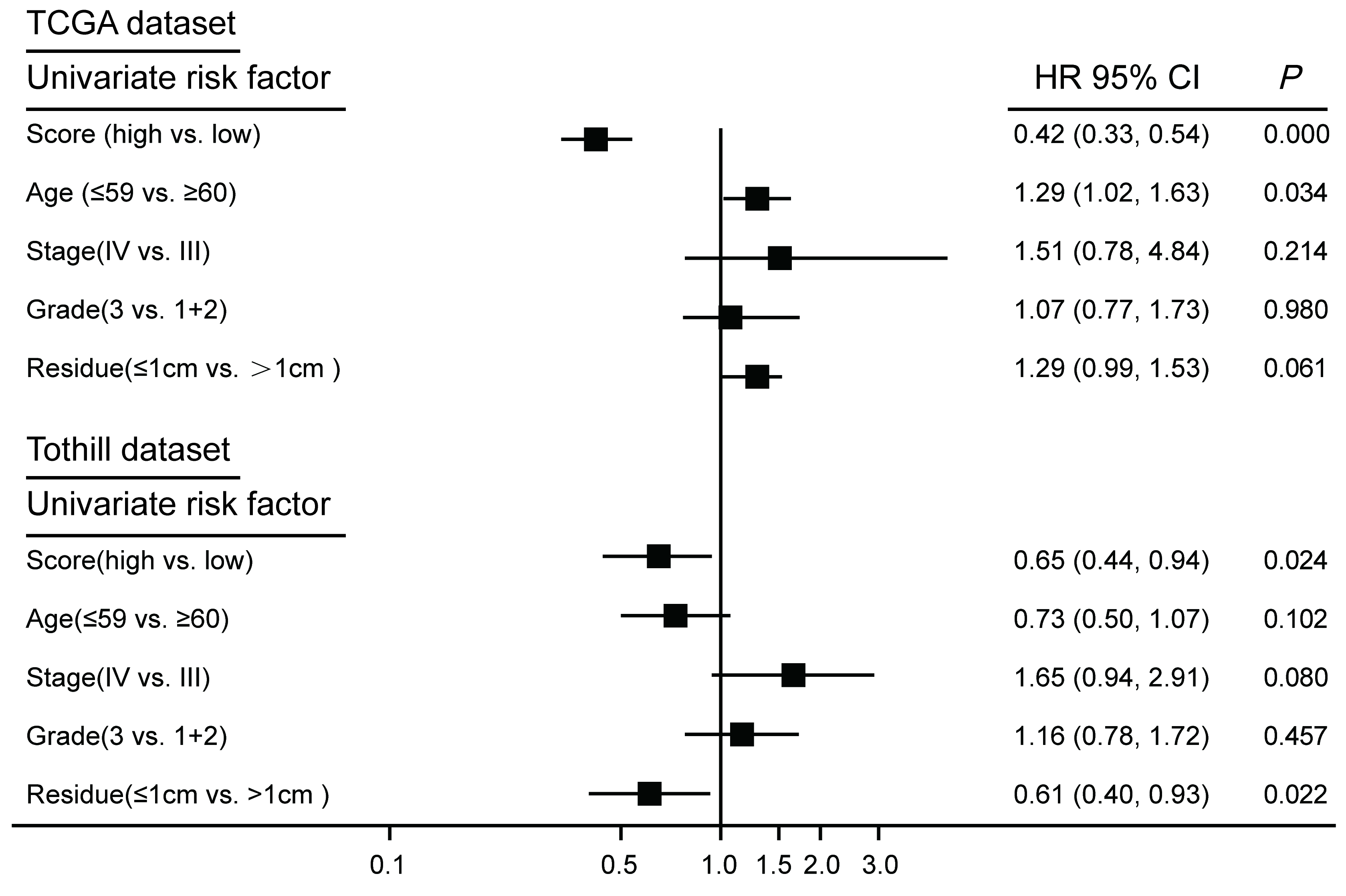

Supplement: Supplementary file 6 — Additional file 6: Figure S4. Univariate analyses were performed in TCGA and Tothill dataset. (tif 1.21mb) Univariate analyses incorporating the score and known prognostic clinical factors, including age at diagnosis (≤59 v.s. ≥60 years), stage (III v.s. IV), grade (1–2 v.s. 3), and surgical debulking (0–10 v.s. ≥11 mm residual tumor). Solid squares represent the hazard ratio (HR) of death and open-ended horizontal lines represent the 95% confidence intervals (CIs). All P values were calculated using Cox proportional hazards analysis. (TIF 1241 kb) [file 12885_2019_6288_MOESM6_ESM.tif]
