# Supplementary material for: A reactive oxygen species scoring system predicts cisplatin sensitivity and prognosis in ovarian cancer patients
Source: BMC Cancer. 2019 Nov 8;19:1061. doi: 10.1186/s12885-019-6288-7 (PMC6839150; doi:10.1186/s12885-019-6288-7)
Supplement: Supplementary file 3 — Additional file 3: Table S2. The IC50 of ovarian cancer cell lines and primary cancer cells treated with different combinations of drugs. (docx 18.8 kb) (DOCX 18 kb) [file 12885_2019_6288_MOESM3_ESM.docx]

**Table S2**

|  | cDDP(μM)  (Mean ± 95%CI) | cDDP+VitC(μM)  (Mean ± 95%CI) | cDDP+NAC(μM)  (Mean ± 95%CI) | cDDP+GSH(μM)  (Mean ± 95%CI) | cDDP+PIPER(μM)  (Mean ± 95%CI) | cDDP+PLX(μM)  (Mean ± 95%CI) | | cDDP+PEITC(μM)  (Mean ± 95%CI) | |
| --- | --- | --- | --- | --- | --- | --- | --- | --- | --- |
| C13* | 87.43 (75.19-101.7) | 89.62 (81.09-99.05) | 87.72 (77.84-98.86) | 88.04 (77.94-99.45) | 28.03 (23.90-32.89) | 40.87 (33.07-50.51) | 35.00 (28.41-43.11) | |  |
| Caov3 | 39.41 (32.41-47.92) | 43.85 (35.62-53.98) | 42.32 (35.75-50.09) | 41.51 (35.48-48.55) | 23.92 (19.73-29.00) | 27.70 (20.37-37.66) | 30.33 (22.38-41.09) | |  |
| OV-90 | 37.30 (34.13-40.76) | 57.92 (50.98-65.81) | 52.07 (48.67-55.72) | 47.55 (45.97-49.19) | 23.70 (19.68-28.52) | 29.33 (24.59-34.99) | 27.92 (24.07-32.38) | |  |
| OV2008 | 12.16 (6.04-24.48) | 13.57 (9.45-19.49) | 13.23 (7.32-23.92) | 13.43 (7.54-23.95) | 5.94 (2.35-15.03) | 7.45 (3.46-16.04) | 6.10 (2.37-15.73) | |  |
| OVCAR3 | 30.13 (22.71-39.98) | 44.40 (35.32-55.81) | 39.00 (31.51-48.27) | 35.99 (29.25-44.28) | 22.17 (17.69-27.78) | 24.56 (20.78-29.02) | 25.27 (20.83-30.67) | |  |
| SKOV3 | 7.92 (6.14-10.21) | 10.38 (8.94-12.06) | 9.86 (8.58-11.32) | 9.56 (7.83-11.67) | 4.04 (2.83-5.76) | 4.73 (4.00-5.60) | 4.75 (3.76-6.01) | |  |
| Patient1 | 47.42 (42.44-52.98) | 55.07 (50.29-60.31) | 51.56 (46.50-57.16) | 52.61 (47.41-58.37) | 25.83 (23.40-28.52) | 30.49 (28.84-32.24) | 35.25 (32.18-38.60) | |  |
| Patient2 | 43.80 (32.11-59.75) | 49.10 (42.62-56.56) | 47.17 (39.43-56.45) | 45.21 (36.61-55.84) | 24.98 (23.90-26.11) | 28.33 (25.05-32.04) | 31.36 (26.66-36.89) | |  |
| Patient3 | 27.05 (19.17-38.16) | 29.80 (19.41-45.74) | 29.09 (20.36-41.55) | 32.45 (22.25-47.32) | 13.18 (11.04-15.74) | 15.08 (11.89-19.12) | 15.56 (11.96-20.24) | |  |
| Patient4 | 25.00 (23.85-26.21) | 35.89 (25.58-50.36) | 28.87 (22.26-37.44) | 31.13 (24.83-39.04) | 18.36 (14.57-23.14) | 15.90 (13.82-18.30) | 14.32 (13.90-14.76) | |  |
| Patient5 | 21.64 (17.49-26.77) | 27.37 (24.03-31.17) | 34.91 (24.03-50.71) | 33.65 (24.51-46.20) | 12.59 (11.39-13.91) | 13.98 (12.43-15.72) | 15.45 (12.06-19.79) | |  |
| Patient6 | 30.4 (24.61-37.55) | 34.71 (27.87-43.24) | 37.36 (29.01-48.12) | 34.39 (25.20-46.94) | 17.27 (15.45-19.31) | 19.91 (15.59-25.43) | 21.26 (16.16-27.98) | |  |

The IC50 of ovarian cancer cell lines and primary cancer cells treated with different combinations of drug
